# Systemic and Local Administration of Antimicrobial and Cell Therapies to Prevent Methicillin-Resistant* Staphylococcus epidermidis*-Induced Femoral Nonunions in a Rat Model

**DOI:** 10.1155/2016/9595706

**Published:** 2016-07-13

**Authors:** Arianna B. Lovati, Lorenzo Drago, Marta Bottagisio, Matilde Bongio, Marzia Ferrario, Silvia Perego, Veronica Sansoni, Elena De Vecchi, Carlo L. Romanò

**Affiliations:** ^1^Cell and Tissue Engineering Laboratory, IRCCS Galeazzi Orthopaedic Institute, 20161 Milan, Italy; ^2^Laboratory of Clinical Chemistry and Microbiology, IRCCS Galeazzi Orthopaedic Institute, 20161 Milan, Italy; ^3^Department of Biomedical Science for Health, University of Milan, 20133 Milan, Italy; ^4^Department of Veterinary Medicine (DiMeVet), University of Milan, 20133 Milan, Italy; ^5^MAP Laboratory, Fondazione Filarete, 20139 Milan, Italy; ^6^Laboratory of Experimental Biochemistry & Molecular Biology, IRCCS Galeazzi Orthopaedic Institute, 20161 Milan, Italy; ^7^Department of Reconstructive Surgery of Osteoarticular Infections, CRIO Unit, IRCCS Galeazzi Orthopaedic Institute, 20161 Milan, Italy

## Abstract

*S. epidermidis* is responsible for biofilm-related nonunions. This study compares the response to* S. epidermidis*-infected fractures in rats systemically or locally injected with vancomycin or bone marrow mesenchymal stem cells (BMSCs) in preventing the nonunion establishment. The 50% of rats receiving BMSCs intravenously (s-rBMSCs) died after treatment. A higher cytokine trend was measured in BMSCs locally injected rats (l-rBMSCs) at day 3 and in vancomycin systemically injected rats (l-VANC) at day 7 compared to the other groups. At day 14, the highest cytokine values were measured in l-VANC and in l-rBMSCs for IL-10. *µ*CT showed a good bony bridging in s-VANC and excellent both in l-VANC and in l-rBMSCs. The bacterial growth was lower in s-VANC and l-VANC than in l-rBMSCs. Histology demonstrated the presence of new woven bone in s-VANC and a more mature bony bridging was found in l-VANC. The l-rBMSCs showed a poor bony bridging of fibrovascular tissue. Our results could suggest the synergic use of systemic and local injection of vancomycin as an effective treatment to prevent septic nonunions. This study cannot sustain the systemic injection of BMSCs due to high risks, while a deeper insight into local BMSCs immunomodulatory effects is mandatory before developing cell therapies in clinics.

## 1. Introduction

Open fractures are notorious to be at high risk of bacterial contaminations, mainly supported by the osteosynthesis devices that induce the biofilm development and a delayed bone healing [1].* S. epidermidis* is one of the most involved pathogens in bone infections and nonunions [2] creating a protective niche from antimicrobial treatments [3]. At present, the standard therapy for orthopaedic infections implicates the systemic administration of antibiotics [4]. However, the long-term use of antibiotics, insufficient to reach bacteria within the biofilm matrix, generates a multidrug resistance leading to methicillin-resistant* S. epidermidis* (MRSE) [5]. Moreover, the antibiotic prophylaxis could be inadequate in case of fractures associated with vascular injuries, which reduce the local drug concentration. Hence, alternative prophylaxis strategies need to be assessed not only to prevent bacterial infections but also to support the bone repair. Many antimicrobial agents have been incorporated into biomaterials to be locally delivered [6]. Specifically, a novel bioresorbable hydrogel was* in vitro* and* in vivo* validated as an orthopaedic implant coating and antibiotic slow-releasing delivery to impair the bacterial colonization through an antimicrobial competitive inhibition [7–9]. Nowadays, cell therapies have also been proposed to promote the bone repair [10]. Mesenchymal stem cells (MSCs) are claimed to be one of the most frequently used cell types thanks to their high proliferative ability and easy accessibility. MSCs have unique immunologic features that support their viability and proliferation in nonself environments [11]. The use of allogeneic MSCs may drastically reduce the waiting time to obtain a relevant cell amount for clinical use [12], as supported by orthopaedic clinical trials (ClinicalTrial.gov #NCT02307435 and #NCT01586312). Furthermore, MSCs ability to restrain bacterial infections has been hypothesized having both proangiogenic and immunomodulatory characteristics that promote the release of mediators (cytokines and chemokines) [13, 14]. In a recent study, we provided evidence of dose-dependent MRSE-induced nonunions in rats [15], demonstrating that subclinical orthopaedic infections are diagnosed with a significant delay. Typically, in clinics, the C reactive protein remains the most used biomarker of infection, despite a scarce sensitivity and specificity [16]. To support medical treatments for infections, identifying reliable predictive markers is urgency. Cytokines play an important role during the host response to infections inflammation and tissue repair by recruiting the cell mediated immunity, before any clinical appearance [17]. Importantly, the inflammation associated with fractures induces a precocious cytokine release that is essential during the early stage of bone healing [18]. However, a prolonged release of inflammatory cytokines fails to stimulate the osteogenic differentiation of resident MSCs recruited on the fracture site leading to an impaired healing [18], and the presence of bacteria highly influences the inflammatory cytokines and bone healing.

In the present study, we compare the host response to MRSE-related infections of femoral fractures in rats treated with cell therapies, conventional systemic antibiotic prophylaxis, and antibacterial-coated implant devices. We hypothesize that transplanted MSCs in a nonunion rat model could have benefits on bone healing and prevention of septic nonunion development thanks to MSC immunomodulatory effects. Moreover, we hypothesize a role of circulating cytokines in the MRSE-related infections presuming a different activity, according to the received treatments.

## 2. Materials and Methods

### 2.1. Study Design and Procedures

This study on animals was approved by the Mario Negri Institute for Pharmacological Research (IRFMN) Animal Care and Use Committee (IACUC) (Permit number 06/2014-PR). The IRFMN adheres to the principles set out in the following laws, regulations, and policies governing the care and use of laboratory animals: Italian Governing Law (D.lgs 26/2014; Authorization number 19/2008-A issued March 6, 2008, by Ministry of Health); Mario Negri Institutional Regulations and Policies providing internal authorization for persons conducting animal experiments (Quality Management System Certificate—UNI EN ISO 9001:2008—Reg. number 6121); the NIH Guide for the Care and Use of Laboratory Animals (2011 edition); and EU directives and guidelines (EEC Council Directive 2010/63/UE). The Statement of Compliance (Assurance) with the Public Health Service (PHS) Policy on Human Care and Use of Laboratory Animals has been recently reviewed (9/9/2014) and will expire on September 30, 2019 (Animal Welfare Assurance #A5023-01).

Thirty 12-week-old male Wistar rats (body weight 373.56 ± 24.82 g) (Harlan Laboratories SRL) were used in this study. Briefly, rats were maintained under general anesthesia and received a preoperative intramuscular single injection of cefazolin (30 mg/kg, Cefamezin, Teva) and a subcutaneous treatment with carprofen (5 mg/kg, Rimadyl, Pfizer). The rats were osteotomized on the right femur and the fracture was synthesized with stainless steel plate and bicortical screws (all from Zimmer®, Germany). All animals were injected into the femoral defect with an inoculum of 1 × 10^5^ CFU/30 *µ*L of MRSE strain #GOI1153754-03-14, as validated and widely described in our previous study [15]. Briefly, to prepare the inoculum, a colony of the MRSE strain was cultured into Brain Heart Infusion Broth (BioMérieux) and incubated at 37°C for 16 hours. The bacterial pellet was suspended in sterile saline to obtain a 10 McFarland turbidity equal to about 3 × 10^9^ CFU/mL; then the bacterial suspension was diluted with sterile saline solution to obtain a bacterial load of 1 × 10^5^ CFU/30 *µ*L. The bacterial inocula were confirmed by agar plate counting procedures and stored at 4°C until use.

**Figure 1:**
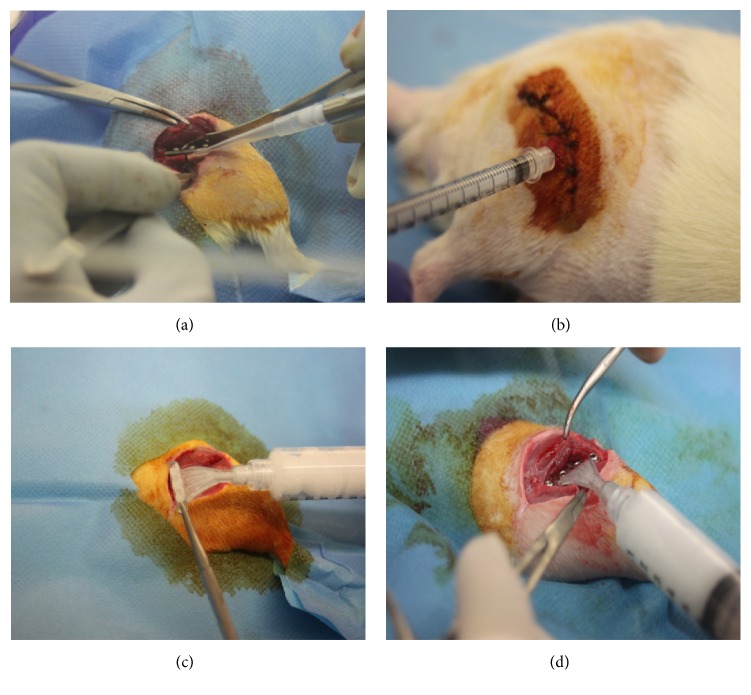
Treatments in the experimental groups. (a) All animals received MRSE locally. The picture represents the injection of the bacterial suspension within the site of the fracture. (b) Representative picture of the local treatment with rBMSCs in the site of the fracture 24 h after surgery. The picture represents the transcutaneous injection of rBMSCs after disinfection within the site of the fracture. (c) The l-VANC group received a vancomycin-enriched hydrogel locally layered on the plate surface before the fracture stabilization and (d) within the site of the osteosynthesis after the plate fixation. The pictures represent the distribution of 250 *µ*L of the vancomycin-enriched hydrogel on the bottom (c) and top side of the plate (d).

After the bacterial inoculum, the muscular planes were closed with Polysorb 4/0 and the skin with Monosof 4/0 (Covidien). The rats were randomly divided into five groups (*n* = 6 each group): the positive control group (PC) did not receive any therapeutic treatment (Figure 1(a)); the systemically treated groups received intravenously vancomycin (15 mg/kg, Hikma) (s-VANC) or allogeneic rat bone marrow MSCs (s-rBMSCs) immediately after surgery; the locally treated groups received a local injection of rBMSCs (l-rBMSCs) (Figure 1(b)) 24 hours after surgery or a local layering of a vancomycin-enriched hydrogel (l-VANC) (Figures 1(c) and 1(d)) during surgery.

Atipamezole (1 mg/kg, Antisedan, Pfizer) was administered subcutaneously to recover rats from general anesthesia. The animals were monitored daily for general status and welfare, clinical signs of infection, lameness, weight bearing, swelling, local hyperemia, wound healing, serous exudate, hematoma, pain, and suffering. The pain was controlled with buprenorphine (0.1 mg/kg SC, Temgesic, Schering Plough, Italy) immediately after surgery.

Three animals of the s-rBMSCs group died within 6–10 hours after surgery for respiratory complications. Their lungs and hearts were explanted and histologically analyzed. From here on, the investigations regarding the s-rBMSCs group were performed on the remaining three animals.

Overall, the animals were monitored for body weight changes, neutrophil counts, and circulating cytokines during the follow-up period. After 6 weeks, rats were euthanized by CO_2_ and *µ*CT scans; microbiological and histological analyses were performed to assess the bone healing and infection.

### 2.2. Culture and Preparation of Rat BMSCs

Allogeneic Wistar rBMSCs (Oricell*™*, Cyagen Biosciences, Cat. number RAWMX-01001, passage 2) were used. Cells were expanded in medium composed of 4.5 g/L glucose Dulbecco's Modified Eagle's Medium, 100 U/mL penicillin-streptomycin, 2 mM L-glutamine, 1% sodium pyruvate, 1% HEPES (all from Gibco), and 10% fetal bovine serum (Hyclone). At passage 5, undifferentiated rBMSCs were differently injected in the rats at concentration of 2 × 10^6^ cells/kg.

### 2.3. Antimicrobial Coating Preparation

A resorbable hydrogel called DAC® (Defensive Antibacterial Coating, Novagenit Srl) was enriched with vancomycin at 5% (v/w), according to manufacturer's guidelines and others [8], and then distributed on plates and screws during the osteosynthesis of the l-VANC group. Briefly, to prepare the vancomycin-enriched hydrogel, 500 mg of vancomycin was diluted in 10 mL of sterile water, and then 5 mL of this suspension was used to solubilize the hydrogel, thus obtaining an enriched hydrogel containing 50 mg/mL of vancomycin. In the l-VANC group, plates and screws implanted were coated with 250 *µ*L of enriched hydrogel, thus delivering locally 35 mg/kg of vancomycin.

### 2.4. Body Weight and Blood Analyses

The rat body weight was measured before surgery and weekly until the day of explantation (day 42) and reported as relative b.w. increase on the baseline (day of surgery). On days 0, 14, and 42 (*n* = 6 per group; *n* = 3 s-rBMSCs), venous blood was harvested from the tail vein under general anesthesia and then transferred into K_2_EDTA tubes (Microtainer MAP, Becton Dickinson) to determine the neutrophil count. On days 3, 7, and 14 after surgery, plasma was obtained by centrifuging the samples (*n* = 4 per group; *n* = 3 s-rBMSCs) at 1200 ×g for 10 min at RT and stored at −80°C until use for the cytokine analysis (IL-1*α*/*β*, IL-6, IL-10, TNF-*α*, and IFN-*γ*) by means of the Luminex assay kit (Bio-Plex Pro*™* Rat Cytokine Assay, Bio-Rad) according to the manufacturer's instructions. Measurement was performed in duplicate by using a Bio-Plex 200 system based on the Luminex xMAP technology (Bio-Rad, Hercules). Cytokine levels are reported as pg/mL.

### 2.5. Micro-CT Imaging

The qualitative and quantitative *µ*CT analyses on femurs were performed with an Explore Locus *µ*CT scanner (GE Healthcare), as previously described elsewhere [15].

Bony bridging percentage of >75%, 50–75%, or <75% of the fracture gap was evaluated and scored. The bone volume (BV, mm^3^) and tissue mineral density (TMD, mg/cc) were calculated within the volume of interest, as described by others [19]. Data were reported as fold increase of the treated groups on the PC group.

### 2.6. Microbiological Analysis

After 42 days, bacteria were recovered from explanted femurs (*n* = 6 per group; *n* = 3 s-rBMSCs) by treating samples with dithiothreitol to dislodge bacteria from the biofilm and analyzed as previously described [15, 20]. Data were reported as Log (CFU/g) explant.

### 2.7. Histological Analysis

Femurs (*n* = 6 per group; *n* = 3 s-rBMSCs) were fixed in 10% formalin, decalcified in Osteodec (Bio-Optica), embedded, and cut into 5 *µ*m sections. Haematoxylin and eosin (H&E) staining was performed to assess morphology, fracture healing, and signs of osteomyelitis. The Gram-positive staining was evaluated for presence or absence of bacteria. Olympus IX71 light microscope and Olympus XC10 camera (Japan) were used to obtain images.

### 2.8. Statistical Analysis

After verifying the normal distribution of data with the Shapiro-Wilk test, comparisons among groups and time points were analyzed with two-way analysis of variance (ANOVA) and comparisons among groups were analyzed with one-way ANOVA (GraphPad Prism v5.00 Software) and then coupled with Bonferroni's* post hoc* test. All data are expressed as means ± standard error (SE). Values of *p* < 0.05 were considered statistically significant.

## 3. Results

### 3.1. Clinical Examination

**Figure 2:**
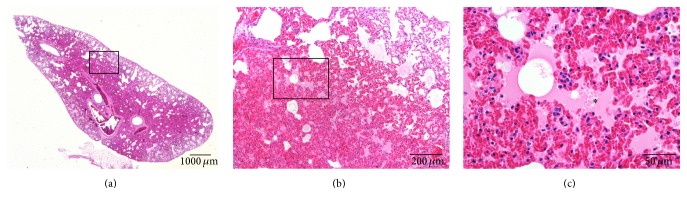
Representative histological panel of the rat lungs reporting the effects of the acute intravenous administration of allogeneic BMSCs in the s-rBMSCs group. The lung sections are stained with haematoxylin and eosin. (a, b) Presence of acute and diffuse hyperemia within the lung parenchyma. (c) Presence of multifocal alveolar edema (*∗*). (a) scale bar 1000 *µ*m; (b) scale bar 200 *µ*m; (c) scale bar 50 *µ*m.

The histological analysis of the organs of the s-rBMSCs treated rats did not show any cardiac alteration but assessed the presence of acute hyperemia associated with multifocal alveolar edema and hemorrhage in the lungs (Figure 2). Despite emboli within pulmonary arteries were not evident, the interlobular septa were markedly inflated with fluid and diffuse congestion (Figure 2, asterisk), presence of macrophages and polymorphonucleated cells within the parenchyma indicating a severe inflammatory reaction.

During the follow-up period, no other animals of any group died or presented peri-implant inflammation. From days 3 to 7, three PC rats showed a partial load bearing on the operated limb without any clinical evidence of infection.

### 3.2. Body Weight and Blood Analyses

**Figure 3:**
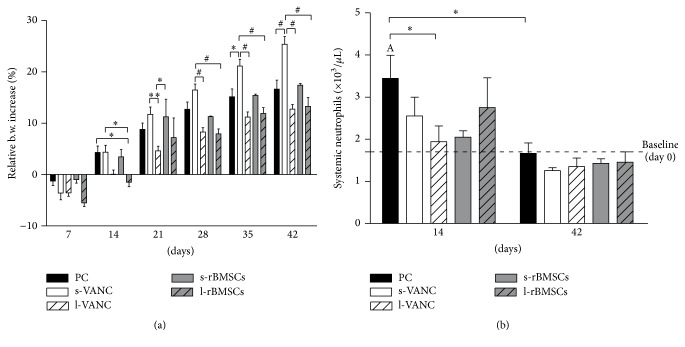
Clinical data. (a) The histogram shows the relative changes in body weight in the experimental groups over time. (b) The histogram shows the systemic neutrophil count in the experimental groups at days 14 and 42 after surgery. Comparisons between groups and time points were analyzed with two-way ANOVA and Bonferroni's* post hoc* test. Statistical significance was *p* < 0.05 (*∗*), *p* < 0.01 (A, *∗∗*), and *p* < 0.001 (#); *n* = 6, *n* = 3 s-rBMSCs.

The relative b.w. increase on the baseline was represented in Figure 3(a). At day 7, a b.w. loss was recorded in all groups. At day 14, l-rBMSCs and l-VANC showed a b.w. decrease and a slower b.w. recovery throughout the experimental follow-up compared to the other treated groups. Differently, s-VANC depicted a significant b.w. increase compared to l-VANC, l-rBMSCs, and PC over time. In Figure 3(b), the neutrophil count is reported as number of neutrophils ×10^3^/*μ*L compared to the baseline (day 0). After 14 days, PC showed a significant neutrophil increase compared to the basal values and to l-VANC.

After 42 days, the neutrophil count almost normalized in all groups without any significant differences compared to the basal values and among the experimental groups.

### 3.3. Pro- and Anti-Inflammatory Cytokines

**Figure 4:**
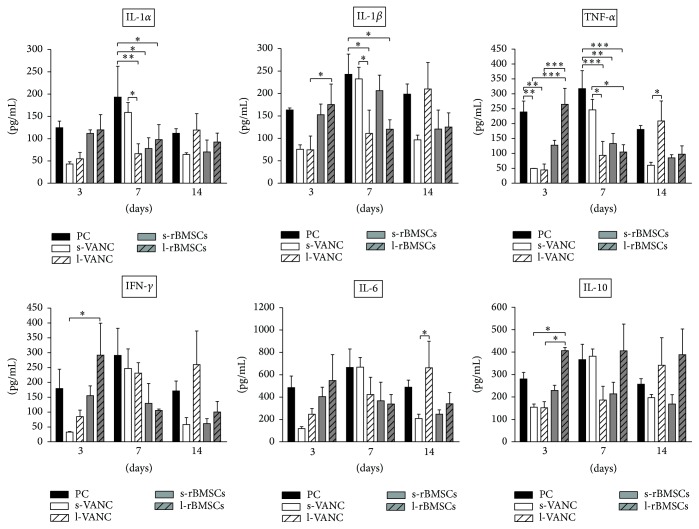
Cytokine analysis. The histograms show the cytokine values of the experimental groups at 3, 7, and 14 days after surgery and bacterial injection. Comparisons between groups and time points were analyzed with two-way ANOVA and Bonferroni's* post hoc* test. Statistical significance was *p* < 0.05 (*∗*), *p* < 0.01 (*∗∗*), and *p* < 0.001 (*∗∗∗*); *n* = 4, *n* = 3 s-rBMSCs.

Plasma levels of proinflammatory (IL-1*α*/*β*, TNF-*α*, and IFN-*γ*), anti-inflammatory (IL-10), and regulatory (IL-6) cytokines were assessed at days 3, 7, and 14 (Figure 4). In most of the groups, all cytokines showed the same changes with time. Overall, at day 3, PC and l-rBMSCs had a higher cytokine trend with respect to the other groups. At day 7, PC and s-VANC showed a higher cytokine trend compared to the other groups. Similarly, this trend was found for IL-1*β* in s-rBMSCs, for IL-10 in l-rBMSCs, and for IFN-*γ* in l-VANC. At day 14, the highest cytokine values were measured in PC and l-VANC and just for IL-10 in the l-rBMSCs group.

Particularly, PC had a higher trend for all the analyzed cytokines compared to the other groups at day 3 and peaked at 7 days after injection. Moreover, PC demonstrated the highest cytokine activity compared to the treated groups. Mainly at day 7, PC had increased values of the acute phase cytokines (IL-1*α*/*β* and TNF-*α*) with significant differences compared to l-VANC, s-rBMSCs, and l-rBMSCs. This trend was found also on day 3 for TNF-*α* in the PC group. Moreover, s-VANC showed a higher trend compared to l-VANC. Differently, the anti-inflammatory cytokines (IL-10) and the late phase (IFN-*γ* and IL-6) did not show a significant difference in PC compared to all the treated groups. At day 3, l-rBMSCs showed higher values for IL-1*α*/*β*, TNF-*α*, and IFN-*γ* with respect to s-VANC and l-VANC. At day 14, l-VANC showed a higher trend with respect to the other groups in all the analyzed cytokines, with a significant difference for TNF-*α* and IL-6 with respect to s-VANC.

### 3.4. Micro-CT Imaging Diagnosis

The *µ*CT qualitative analysis depicted a variable percentage of bony bridging in the experimental groups (Table 1).

**Figure 5:**
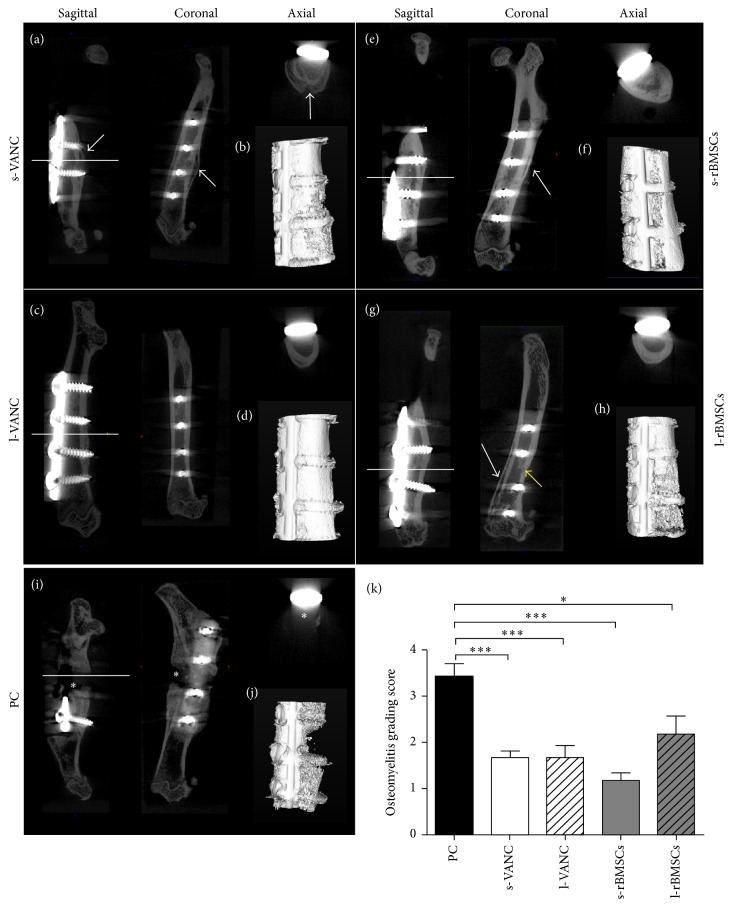
Qualitative *µ*CT imaging, isosurface, and semiquantitative osteomyelitis score. The representative panel shows *µ*CT images on the day of explantation. (a, c, e, g, i) Sagittal, coronal, and axial planes. (b, d, f, h, j) 3D isosurface reconstruction is presented for the s-VANC, s-rBMSCs, l-VANC, l-rBMSCs, and PC groups. Symbols indicate cortical reaction (white arrows); medullary reaction (yellow arrow); and loss of cortical wall and osteolysis (asterisks). (k) Osteomyelitis grading score based on Odekerken's scale is reported in the histogram. Comparisons among groups were analyzed with one-way ANOVA corrected with Bonferroni's* post hoc* test. Statistical significance was *p* < 0.05 (*∗*) and *p* < 0.001 (*∗∗∗*); *n* = 6, *n* = 3 s-rBMSCs.

In particular, s-VANC displayed a good to total bony bridging in the 67% and 33% of the cases, respectively. In this group, no bone osteolysis was detected and a mild cortical reaction was visible near to the fracture site (Figure 5(a), white arrows), confirmed by the 3D reconstruction (Figure 5(b)). Overall, l-VANC (Figures 5(c) and 5(d)), s-rBMSCs (Figures 5(e) and 5(f)), and l-rBMSCs (Figures 5(g) and 5(h)) showed a higher percentage of fracture healing characterized by a well-structured bone callus and mineralized cortices in most of the cases (50 to 100%), and a good osseointegration of the screws was present in all cases. A medullary reaction was detected in l-rBMSCs (Figure 5(g)) together with a mild cortical reaction identified in the 3D reconstruction (Figure 5(h)). Otherwise, the PC group confirmed data obtained in our previous study [15], depicting an evident nonunion associated with a femoral diaphysis deformity and dislocation of the bone stumps due to the loss of implant stability and severe osteolysis (Figures 5(i) and 5(j)). The osteomyelitis grading score for this group showed significant difference with respect to all the treated groups, while no significant differences were found between the treated groups (Figure 5(k)).

**Figure 6:**
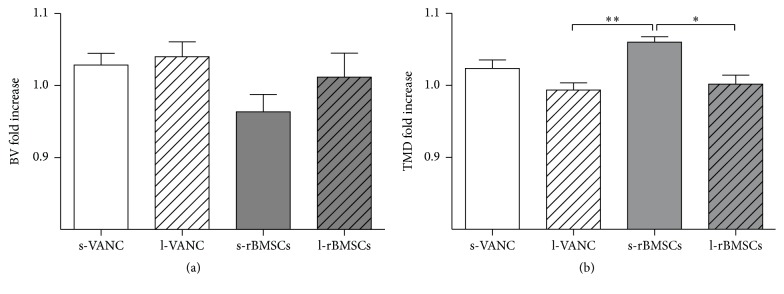
*µ*CT quantitative analyses of bone structure. (a) Bone volume (BV) and (b) tissue mineral density (TMD) quantitative analysis of the treated groups normalized on the PC group, reported as fold increase. Comparisons among groups were analyzed with one-way ANOVA corrected with Bonferroni's* post hoc* test. Statistical significance was *p* < 0.05 (*∗*) and *p* < 0.01 (*∗∗*); *n* = 6, *n* = 3 s-rBMSCs.

BV and TMD were reported as fold increase with respect to the PC group in Figures 6(a) and 6(b). No significant differences were found for BV between the treated groups; differently, the fold increase of TMD was higher in s-rBMSCs with respect to both l-VANC and l-rBMSCs.

### 3.5. Microbiological Analysis

**Figure 7:**
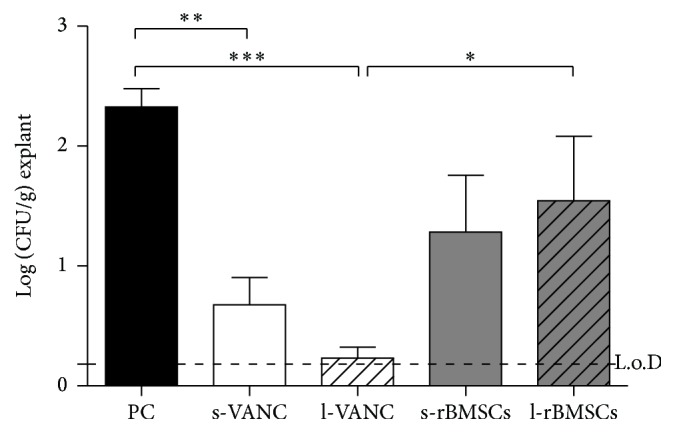
Microbiological detection of bacterial growth on the explanted specimens. The limit of detection (L.o.D.) was set at 0.18 Log (CFU/g)/explant. Comparisons among groups were analyzed with one-way ANOVA corrected with Bonferroni's* post hoc* test. Statistical significance was *p* < 0.05 (*∗*), *p* < 0.01 (*∗∗*), and *p* < 0.001 (*∗∗∗*); *n* = 6, *n* = 3 s-rBMSCs.

The microbiological analysis reported in Figure 7 detected a significant higher bacterial growth between PC and both s-VANC and l-VANC. Moreover, l-VANC showed a lower bacterial growth with respect to l-rBMSCs. The limit of detection was set at 0.18 Log (CFU/g) explant.

### 3.6. Histological Analysis

**Figure 8:**
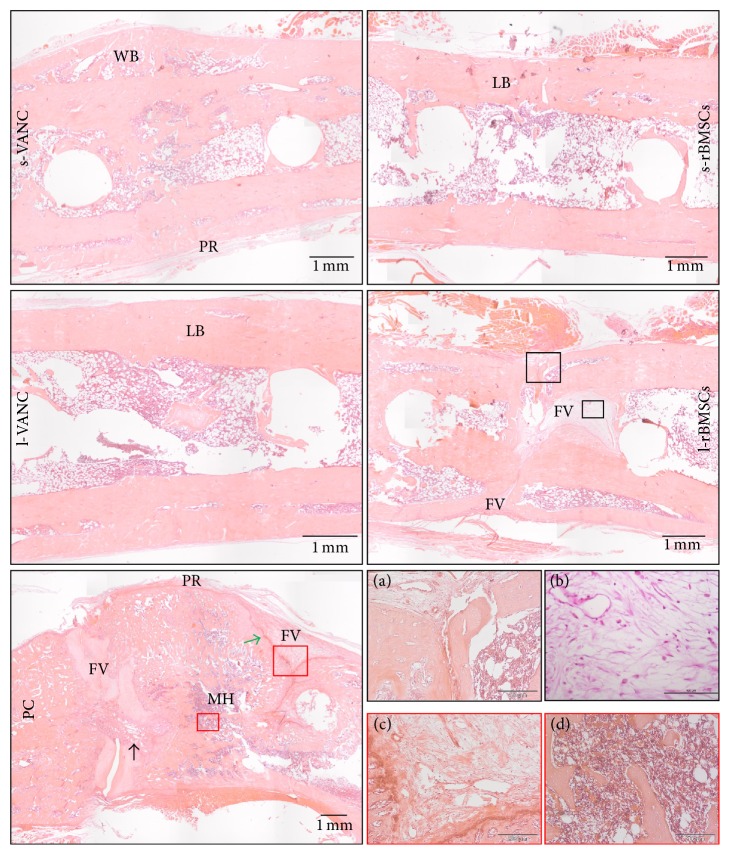
Histological analysis (H&E) at the day of explantation. The representative panel shows H&E staining of the femurs in all the experimental groups. The panels depict an overview of the samples, scale bar 1 mm. WB: woven bone; LB: lamellar bone; PR: periosteal reaction; FV: fibrovascular tissue; MH: myeloid hyperplasia; vascular infiltrates (black arrow); alteration of cortical bone (green arrow). For the l-rBMSCs group, (a) a specific area containing fibrovascular tissue and polymorphonucleated cells is reported in the big black box (scale bar 200 *µ*m) and (b) the presence of giant cells in the small black box (scale bar 100 *µ*m) is reported. For the PC group, (c) a specific area with fibrovascular tissue is reported in the big red box (scale bar 200 *µ*m) and (d) the myeloid hyperplasia is shown in the small red box (scale bar 200 *µ*m).

The H&E staining confirmed the results obtained by *µ*CT in terms of percentage of fracture healing and absence of osteomyelitis in s-VANC, l-VANC, and s-rBMSCs (Figure 8). Specifically, in s-VANC, the fractures appeared repaired by a great amount of newly bone deposition in a remodeling phase (woven bone), coupled with a mild cortical thickening and periosteal reaction. Both in l-VANC and s-rBMSCs, a complete closure of the fracture was found and the new bone appeared more mature and lamellar than in s-VANC. The l-VANC group showed uniformly enlarged cortices with areas of bone remodeling. In l-rBMSCs, 17% of samples presented only a partial bony bridging characterized by a great deposition of fibrovascular tissue invading the fracture site and surrounding the screws disseminated with giant cells. In all the aforementioned groups, a moderate presence of polymorphonucleated cells was found within the medullary canal. The PC group showed disorganized bone architecture, a lot of fibrovascular tissues, and nonunion establishment. The periosteal reaction, myeloid hyperplasia, and presence of intact and fragmented polymorphonuclear cells in the granulation tissue associated with several vascular vessels represented signs of osteomyelitis.

**Figure 9:**
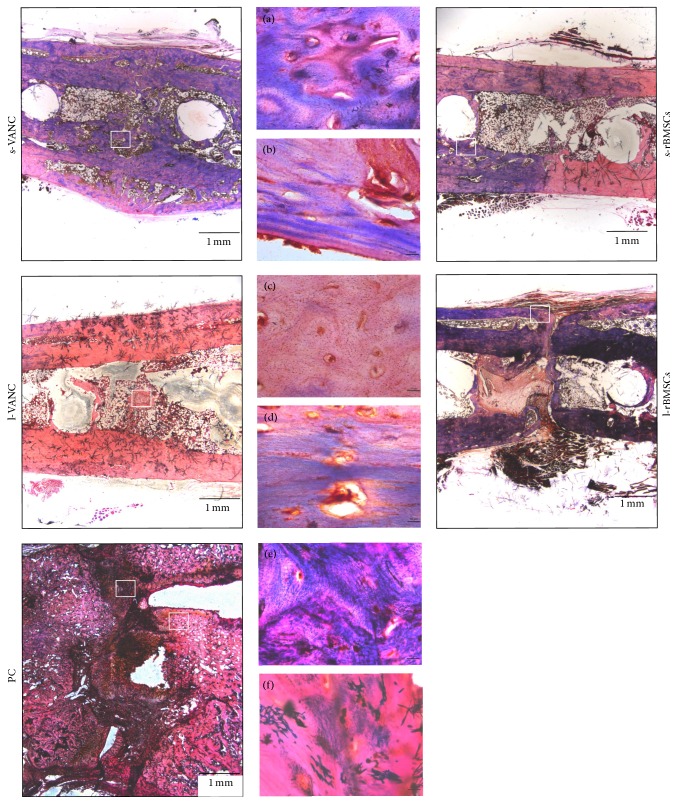
Gram staining of the femurs at the day of explantation. The representative panel shows the Gram staining of the femurs in all the experimental groups. The panels depict an overview of the samples, scale bar 1 mm. In each group, the white boxes have a scale bar 10 *µ*m and represent (a) detail of the s-VANC group; (b) detail of the s-rBMSCs group; (c) detail of the l-VANC group; (d) detail of the l-rBMSCs group; (e, f) details of the PC group.

The Gram staining confirmed the quantitative data obtained from the microbiological tests (Figure 9). Indeed, in all groups, the presence of cocci was detected. In particular, s-VANC, s-rBMSCs, l-rBMSCs, and PC showed several cocci assembled in clusters and diffuse within the bone and periosteal tissue. Differently, l-VANC showed scarce dispersed cocci within areas of new bone formation in the fracture site.

## 4. Discussion

This comparative study analyzes for the first time the efficacy of systemic antimicrobial prophylaxis and antibacterial coating of orthopaedic implants and cell therapy on MRSE-induced nonunions in rats. We hypothesized that the use of allogeneic MSCs could improve the host response to bacterial infections based on the potential immunomodulatory effects directly on the injured site [21].

In our study, the amount of systemically or locally injected rBMSCs was concordant with the dosage used in the literature for cardiovascular or autoimmune diseases [22, 23], as demonstrated to reach damaged sites. However, in our series, we had a 50% of animal death when intravenously injected with rBMSCs (s-rBMSCs). The histological analysis supported the “pulmonary first-pass theory,” in which a scarce cellular delivery has been demonstrated due to the lung filter in either animals or humans [24–27]. This phenomenon should be related to the cell adhesion, the activation of the coagulation pathway, and anaphylactic reactions promoted by the allogeneic MSCs causing pulmonary embolisms [23, 28]. Hereon, it is worth taking into account that the data obtained in the s-rBMSCs group considered only three animals and they cannot offer a good sample sizing to properly sustain our results, representing a limit of our study.

The pathogenesis of bone infections after severe fractures is strictly related to the biofilm formation, making difficult both the diagnosis and the efficacy of treatments. Thus, identifying specific biomarkers would be crucial to early detection of the grade of infection. In our study, we evaluated cytokines produced in both the acute (IL-1*α*/*β* and TNF-*α*) and the chronic phases (IFN-*γ*, IL-10, and IL-6) of inflammation/infection and involved in the bone remodeling (IL-6). Specifically, IL-6 has a bivalent function depending on the mode of expression: persistently high (even moderate) levels are associated with a proinflammatory activity whilst a peaking behavior is associated with an anti-inflammatory proregenerative effect [29]. However, the inability to discriminate between changes from postsurgical trauma and MRSE-induced infection could represent a limitation. Overall, we demonstrated significant differences among groups after 7 days of infection. Due to the staphylococcal toxin release, TNF-*α* showed difference already at day 3, being the primary involved cytokine [30]. Moreover, we demonstrated the simultaneous activation of both pro- and anti-inflammatory cytokines.

At day 7, higher IL-1*α*/*β* and TNF-*α* values in the PC group with respect to the others may have been in response to inflammatory stimuli because of bacterial growth and biofilm formation. This correlates with the greater neutrophil count at 14 days, when neutrophils mediate the recruitment of macrophages maintaining high levels of cytokines [31, 32]. Similarly, on day 7, s-VANC showed increased IL-1*α*/*β* and TNF-*α* values compared to the other treated groups, relating to a reduced efficacy of one-shot injected vancomycin. It is known that vancomycin acts both directly against bacteria and as an immunomodulatory drug, inhibiting the cytokine production during the early stages [33], as we also demonstrated at day 3.

On day 14, higher cytokine levels in l-VANC compared to the other groups could be caused by the local inflammatory response through the activation of macrophages that intervene against the material debris and could affect the bone repair, as also demonstrated by others [34]. Moreover, we detected high levels of IL-6 in l-VANC after 14 days, predicting a lower systemic efficacy of this treatment with respect to the conventional prophylaxis therapy [35]. However, IL-6 is involved in the modulation of bone cells during repair by suppressing the differentiation of the osteoclast progenitors [36, 37]. The increase of IL-1*β*, IFN-*γ*, and TNF-*α* found in l-rBMSCs at day 3 could be related to the antiapoptotic activity of MSCs on neutrophils in the microenvironment of a damaged and infected tissue [38, 39]. These data were also confirmed by the neutrophil analysis and were consistent with those described by Seebach et al. [13]. Furthermore, these inflammatory cytokines can stimulate the MSCs to release a large amount of growth factors promoting the tissue repair [40]. Again, we detected an upregulation of IL-10 in l-rBMSCs at days 7 and 14. Specifically, IL-10, produced by the macrophages present in the histological sections of l-rBMSCs, has a regulatory role in immunological and inflammatory responses by decreasing the production of proinflammatory cytokines, as demonstrated here and by others [41]. Indeed, the interaction between MSCs and macrophages secretes prostaglandin E2 that reprograms the macrophages to release IL-10 [39]. The increase of IL-10 in l-rBMSCs suggests that MSCs can modulate the host immune response to infection. Otherwise, a similar behavior for the s-rBMSCs group could not be supported by this study because of the small number of survived animals. Thus, the clinical results in the s-rBMSCs group (*µ*CT and histology) cannot be considered representative for the efficacy of this treatment. Concerning the other groups, the semiquantitative Odekerken's score for osteomyelitis was supported by the BV and TMD measurements, in which no significant differences were found among s-VANC, l-VANC, and l-BMSCs groups.

The *µ*CT and histological analyses of the PC group generated results consistent with those of our previous study [15], demonstrating the development of septic nonunions. The same analyses highlighted a worse osteomyelitis score in the l-rBMSCs group compared to the antibiotic treated groups, as also supported by Seebach et al. [13]. This is potentially due to the release of cellular proteases by dead MSCs that could negatively act on bone repair and support the bacterial colonization. This was also demonstrated by the microbiological tests measuring a greater bacterial growth in l-rBMSCs compared to the antibiotic treated groups.

Overall, our study demonstrated a good response in terms of bone healing and absence of osteomyelitis in s-VANC and l-VANC.

## 5. Conclusions

Through our results, we could suggest the synergic use of systemically injected vancomycin and its local delivery as an effective treatment to prevent the bacterial spread in orthopaedic infections. The hydrogel, used in this study, could also ameliorate the bone repair towards a more mature bone thanks to its capability in stimulating bone specific cytokine (IL-6). Otherwise, our study cannot definitely sustain the use of cell therapy for this purpose. Indeed, the intravenous injection of MSCs should be considered a highly risky treatment with a high rate of mortality. However, based on our preliminary results on the local injection of MSCs, a deeper insight into their immunomodulatory mechanisms in a large experimental design should be helpful to develop novel strategies for the clinical use of MSCs.

## Figures and Tables

**Table 1:** Percentage of bony bridging of the fracture site.

	Bony bridging < 75% nonunion fracture	Bony bridging 50–75% partial fracture healing	Bony bridging > 75% fracture healing
PC	83% (5/6)	17% (1/6)	n.d. (0/6)
s-VANC	n.d. (0/6)	67% (4/6)	33% (2/6)
l-VANC	33% (2/6)	17% (1/6)	50% (3/6)
s-rBMSCs	n.d. (0/3)	n.d. (0/3)	100% (3/3)
l-rBMSCs	33% (2/6)	17% (1/6)	50% (3/6)

n.d.: nondetectable.
